# Highly efficient organic light emitting diodes formed by solution processed red emitters with evaporated blue common layer structure

**DOI:** 10.1038/srep15903

**Published:** 2015-10-30

**Authors:** Ye Ram Cho, Hyung Suk Kim, Young-Jun Yu, Min Chul Suh

**Affiliations:** 1 Department of Information Display, Kyung Hee University, 26 Kyungheedae-ro, Dongdaemun-gu, Seoul 02447, Korea; 2 OLED Advanced Technology Team 1, LG Display, Paju-si, Gyeonggi-do 10844, Korea

## Abstract

We prepared highly-efficient solution-processed red phosphorescent organic light emitting diodes (PHOLEDs) with a blue common layer structure that can reasonably confine the triplet excitons inside of the red emission layer (EML) with the assistance of a bipolar exciton blocking layer. The red PHOLEDs containing EML with a 7 : 3 ratio of 11-(4,6-diphenyl-[1,3,5]triazin-2-yl)-12-phenyl-11,12-dihydro-11,12-diaza-indeno[2,1-*a*]fluorene (n-type host, NH) : 4-(3-(triphenylen-2-yl)phenyl)dibenzo[*b,d*]thiophene (p-type host, PH) doped with 5% Iridium(III) bis(2-(3,5-dimethylphenyl)quinolinato-*N,C2’*)tetramethylheptadionate (Red Dopant, RD) produced the highest current and power efficiencies at 23.4 cd/A and 13.6 lm/W, with a 19% external quantum efficiency at 1000 cd/m^2^. To the best of our knowledge, such efficiency was the best among those that have been obtained from solution-processed small molecular red PHOLEDs. In addition, the host molecules utilized in this study have no flexible spacers, such as an alkyl chain, which normally deteriorate the stability of the device.

Over the past few decades, organic light-emitting diodes (OLEDs) have been of great interest for use in displays due to their superior image quality resulting from the self-emissive property, low power consumption, wide viewing angle, wide color gamut, high-speed video rate, etc^1^,^2^. However, active matrix organic light emitting diodes (AMOLED) have encountered enormous difficulties in meeting the low-cost, high-resolution requirements for large-sized OLED devices due to a limitation in vacuum thermal evaporation technology. Therefore, a solution process has drawn a considerable amount of attention as an alternative for production because it allows low-cost, large-area processing^3^,^4^. Unfortunately, this process has very critical limitations in terms of the material properties. In other words, soluble materials are not yet adequate for use in display applications, especially as a result of the seriously short lifetime of the emitters because soluble emitters are normally designed to contain alkyl chains in order to improve their solubility by increasing the free volume^5^. However, the introduction of alkyl chains generally has a negative impact on the long-term stability, that is, the lifetime. Hence, numerous studies have been conducted to overcome such performance limitations in devices fabricated through a solution process. The most important way to improve performance, including the long-term stability of OLED devices, is to obtain a good charge balance by using various types of layers, such as a hole injection layer (HIL), hole transport layers (HTL), electron blocking layer (EBL), emitting layers (EMLs), hole blocking layer (HBL), electron transport layer (ETL), electron injection layer (EIL), etc.^6^,^7^,^8^. In this aspect, it is really difficult to obtain highly efficient and stable devices with a perfect charge balance because the multi-layer stacking of the functional materials by solution process is almost impossible. The main reason for such an issue could presumably be a result of the lack of materials with perfect orthogonality on the solubility, which typically causes a mixing of the polymeric or small molecular species at the interface of the functional layers that comprise such materials during the successive soluble processes^9^,^10^,^11^,^12^,^13^.

So far, the “advanced hybrid device structure approach” which allows limited numbers of solution processable materials is the representative method to achieve reasonable device performances for commercialization. Meanwhile, the utilization of a blue common layer which normally deposit the blue emitter on the patterned red and green EMLs^14^,^15^,^16^ is very helpful to obtain the reasonable performances including lifetime because the soluble blue emitter has extremely short lifetime. In other words, we could ink-jet print HIL, HTL, red and green EMLs, and then successively deposit a bipolar exciton blocking layer (B-EBL), blue EML, ETL, EIL, and cathode by using a thermal evaporation process. Among the many functional layers, B-EBL plays an important role in suppressing the triplet exciton migration toward the blue EML in the structure^17^. Nevertheless, the performance of the solution-processed red and green emitters is still poorer than that of emitters prepared with a vacuum thermal evaporation process. Thus, many reports have attempted to improve the performance of devices fabricated with solution processes^18^,^19^. One of the most important methods to improve the performance is to broaden the recombination zone by applying mixed host systems^20^,^21^. Second, we could also improve the device properties by using exciton blocking layers with a much higher triplet energy (T_1_) (by ~0.2 eV) compared to that of the phosphorescent dopant in the EMLs. Third, we must obtain a film with excellent morphology after the spin coating or ink-jet printing processes. Of those methods, the mixed host approach is also being used to form green emitters in modern mass production of AMOLED devices by using an evaporation process. Interestingly, such an approach often causes problems, such as a change in the composition of the EML during the evaporation process. In particular, there are not so many options to select such materials because they normally mix the host materials with a similar vapor pressure and evaporate them all together to prevent the unwanted changes in composition mentioned above. In this respect, the solution-processed OLEDs may have a superior advantage in that they provide plentiful material choice because we could control the composition of the mixed host system with precision in the EML solution. Nevertheless, the poor solubility of the small molecular host materials for this application is still problematic because they show poor solubility if they have no alkyl substituent that normally reduces the stability of the OLEDs.

In this paper, we report on highly-efficient solution-processed red PHOLEDs (phosphorescent OLEDs) by using a relatively good solubility without any alkyl group. We fabricated the devices by using an advanced hybrid device structure. In addition, we also utilized a mixed host system to improve the device performance.

## Results

**Figure 1:**
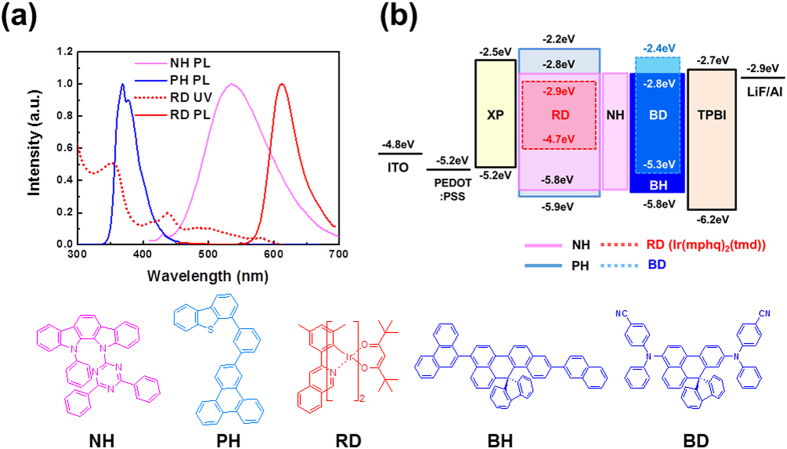
(**a**) UV-Visible absorption spectrum of dopant (RD) and photoluminescence spectra (at 298 K) of dopant (RD) and host materials (NH and PH) in tetrahydrofuran (THF). (**b**) Device structure and energy band diagram of solution processed red PHOLED.

Figure 1(a) shows the UV-Vis (Ultraviolet-Visible) absorption spectrum of the dopant (dashed line) and the photoluminescence (PL) spectra of the hosts and dopant (solid line) in tetrahydrofuran (THF). An effective energy transfer process requires a spectrum overlap between the absorption spectrum of the dopant material and the emission spectrum of the host material. As shown in Fig. 1(a), the spectral range and the intensity of the absorption spectrum of Iridium(III) bis(2-(3,5-dimethylphenyl)quinolinato-*N,C2’*)tetramethylheptadionate (Red Dopant, RD) is very broad and is moderately strong at a range below 610 nm, so it overlaps properly with the PL spectra of 11-(4,6-diphenyl-[1,3,5]triazin-2-yl)-12-phenyl-11,12-dihydro-11,12-diaza-indeno^2^ fluorene (n-type host, NH) and 4-(3-(triphenylen-2-yl)phenyl)dibenzo[*b,d*]thiophene (p-type host, PH), which shows a very broad emission between 420 and 700 nm in the case of NH and a slightly more narrow spectral range between 340 and 450 nm in the case of PH. Thus, the energy transfer could be very efficient within these spectral ranges.

Meanwhile, the bipolar characteristics of the host materials with proper triplet energy levels are very important to improve the efficiency because they help broaden the exciton recombination zone inside of the emission layer of the PHOLEDs. In addition, the charge balance at the emission layer is controlled with precision and is also very important in improving the stability and efficiency of the devices. The relative charge carrier transport ability of the EML was investigated by preparing hole-only devices (HODs) as well as electron-only devices (EODs) of NH and PH as n-type and p-type host materials^22^,^23^. We used molybdenum oxide (MoO_3_) and lithium quinolate (LiQ) as charge carrier injection layers for indium tin oxide (ITO) and aluminum (Al) as follows^24^:

HOD A: ITO/MoO_3_ (0.75 nm)/NH (100 nm)/MoO_3_ (10 nm)/Al

HOD B: ITO/MoO_3_ (0.75 nm)/PH (100 nm)/MoO_3_ (10 nm)/Al

EOD A: ITO/LiQ (1.5 nm)/NH (100 nm)/LiQ (1.5 nm)/Al

EOD B: ITO/LiQ (1.5 nm)/PH (100 nm)/LiQ (1.5 nm)/Al

**Figure 2:**
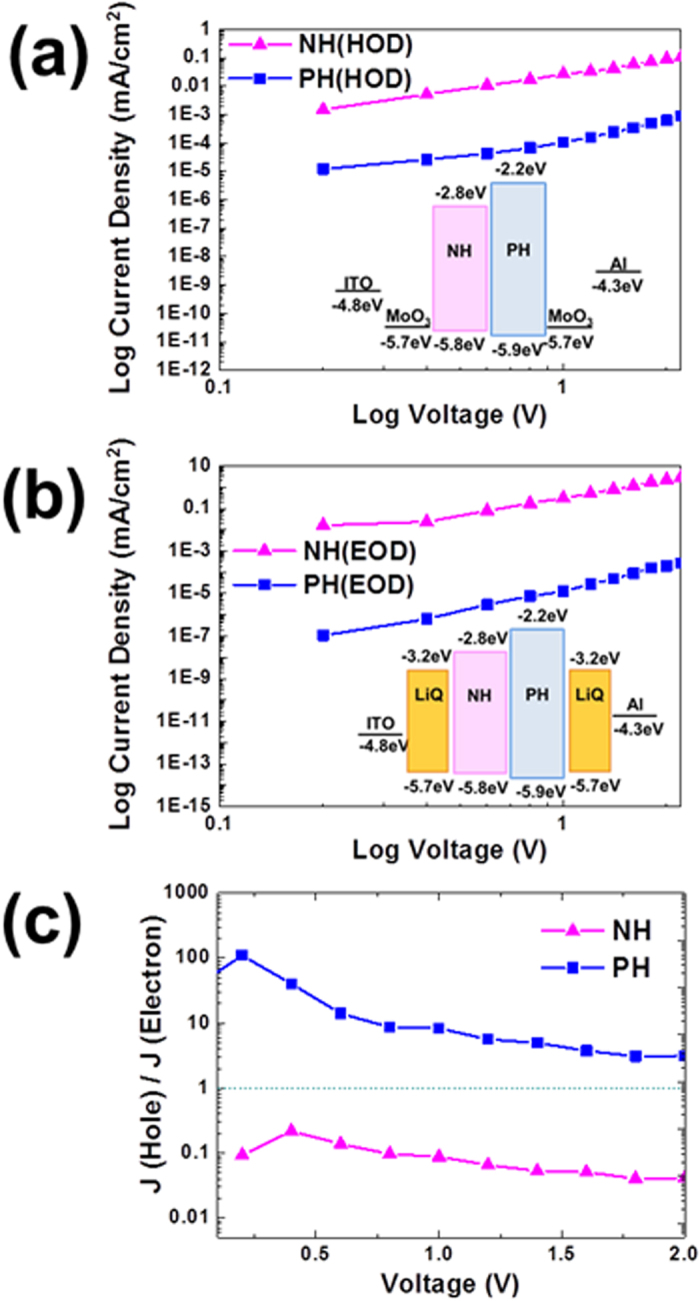
*J-V* characteristics of (a) HOD and (b) EOD fabricated in this study and (c) relative current density behavior of host materials.

The inset of Fig. 2(a,b) shows the energy band diagram of the HODs and EODs of the host materials that were used in this study. 0.75 nm of MoO_3_ were deposited on the front of the host materials for the HOD devices. MoO_3_ helps attain an effective ohmic injection of the hole carriers into the Highest Occupied Molecular Orbital (HOMO). A 10 nm thick MoO_3_ layer deposited after the host materials to block the flow of electrons from the cathode to the Lowest Unoccupied Molecular Orbital (LUMO) of the host materials. At the EODs, LiQ/Al were used to inject the electron into the LUMO of host materials because it has also been widely known to be an effective cathode system towards general ETL materials. The current density - voltage (*J*–*V*) characteristics that were obtained from the HODs and EODs mentioned above are shown in Fig. 2(a,b). Our results suggest that NH causes significant levels of hole current as well as electron current while the PH make much less hole and electron current flow through the bulk of the material. We plotted the relative current density of those devices to estimate the charge balance when the materials are used as host materials in the emission layer by dividing the hole current density of the HODs by the electron current density of the EODs [e.g. *J*(hole)/*J*(electron) where *J*: current density, mA/cm^2^] as shown in Fig. 2(c). From this plot, we could expect that the NH generates a much higher electron current density (by >10 times) while the PH produces a moderately higher hole current density (by 2–5 times). Thus, we could select the best composition of the NH and PH by monitoring the relative current density values, whether it is close to unity or not, by mixing such host materials. However, it is really hard to expect such an ideal mixing ratio just by investigating the relative current density of the devices because there are many other factors that affect the characteristics of solution processed devices, such as the film morphology, solubility, etc.

**Figure 3:**
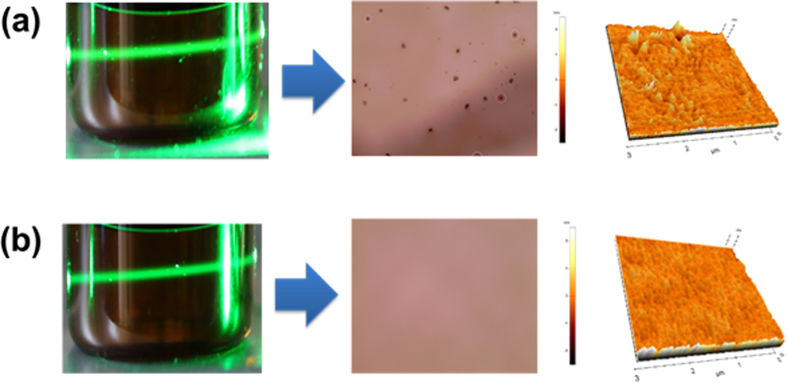
The photographs of the organic solutions in chlorobenzene to investigate Tyndall effect, resultant surfaces after spin casting and AFM images of EML surfaces formed by spin coating process from (a) PH and (b) NH solutions, respectively.

Hence, we must investigate the solubility of the small molecular solution through a Tyndall test before we prepare the solution-processed devices. In other words, we could obtain the solubility information for certain small molecules by exposing the green laser through a small molecular solution, as shown in Fig. 3. We can use this method to judge that the PH provides a slightly inhomogeneous solution in chlorobenzene because we could detect the relatively diffusive and hazy laser trace through a chlorobenzene solution of such material, as shown in the picture of the left-hand side of Fig. 3(a). As a result, we could not obtain a good film morphology, as shown in the picture of the middle side of Fig. 3(a). In contrast, the laser trace in the case of the NH solution was more or less clearer than that from the PH solution, as shown in the left-hand side of Fig. 3(b). Similarly, we obtained a film with a perfect morphology from this coating solution [see middle side of Fig. 3(b)].

We confirmed the morphology of such layers by preparing samples containing poly(3,4-ethylene dioxythiophene) (PEDOT) doped with poly(styrene sulfonate) (PSS) (PEDOT:PSS) and HL-X026, which is a crosslinking polymer (XP, T_1_: 2.25 eV) obtained from Merck, as a hole transporting material^25^,^26^ beneath, and the surface profile images were obtained via AFM (Atomic Force Microscope), as shown in Fig. 3. The right-hand side of Fig. 3(a) is the surface morphology of the PH layer. The root-mean-square roughness (RMS) value of the PH films was of about 12.6 Å. On the other hand, the NH film provided a much smoother film with an RMS value of 6.2 Å. This means that the PH cannot provide reliable device characteristics when compared to the NH materials when they were spun cast on the pre-coated surface with HIL/HTL. Thus, we prepared five different red PHOLEDs with the basic structures as follows. [The energy diagram is shown in Fig. 1(b).]

Device A: ITO/PEDOT:PSS (40 nm)/XP (17 nm)/NH:PH:RD (0:10, 5%, 30 nm)/NH (5 nm)/BH:BD (5%, 15 nm)/TPBI (20 nm)/LiF (0.5 nm)/Al (100 nm).

Device B: ITO/PEDOT:PSS (40 nm)/XP (17 nm)/NH:PH:RD (3:7, 5%, 30 nm)/NH (5 nm)/BH:BD (5%, 15 nm)/TPBI (20 nm)/LiF (0.5 nm)/Al (100 nm).

Device C: ITO/PEDOT:PSS (40 nm)/XP (17 nm)/NH:PH:RD (5:5, 5%, 30 nm)/NH (5 nm)/BH:BD (5%, 15 nm)/TPBI (20 nm)/LiF (0.5 nm)/Al (100 nm).

Device D: ITO/PEDOT:PSS (40 nm)/XP (17 nm)/NH:PH:RD (7:3, 5%, 30 nm)/NH (5 nm)/BH:BD (5%, 15 nm)/TPBI (20 nm)/LiF (0.5 nm)/Al (100 nm).

Device E: ITO/PEDOT:PSS (40 nm)/XP (17 nm)/NH:PH:RD (10:0, 5%, 30 nm)/NH (5 nm)/BH:BD (5%, 15 nm)/TPBI (20 nm)/LiF (0.5 nm)/Al (100 nm).

We deposited 5 nm of NH (T_1_ : 2.82 eV)^25^ as a B-EBL to protect the exciton quenching at the blue EML (T_1_ : 2.50 eV)^26^ although the blue EML used for this study is reasonable high to confine the excitons inside EML as previously discussed^17^. Then, we continuously evaporated blue EML with 10-(naphthalen-2-yl)-3-(phenanthren-9-yl)spiro[benzo[*ij*] tetraphene-7,9’-fluorene] as a blue host material (BH), *N*^*6*^*,N*^*9*^-bis(4-cyanophenyl)-*N*^*3*^*,N*^*9*^-diphenylspiro[benzo[*de*]anthracene-7,9’-fluorene]-3,9-diamine as a blue dopant (BD)^26^, 2,2’,2”-(1,3,5-phenylene)*tris*(1-phenyl-*1H*-benzimidazole) (TPBI) as an electron transporting material, lithium fluoride (LiF) as an electron injection layer (EIL), and Al as a cathode.

## Discussion

**Figure 4:**
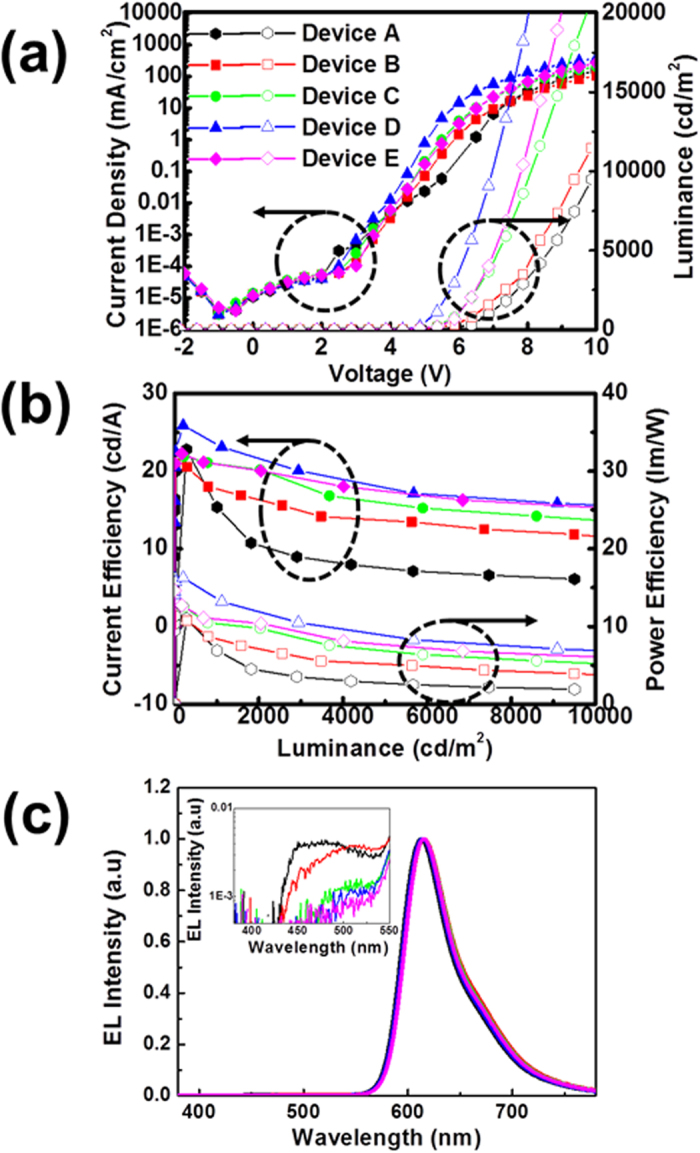
Device characteristics of fabricated red PHOLEDs in this study (a) *J-V-L* and (b) Luminance-efficiency curves and (c) normalized EL (electroluminescent) spectra of fabricated red PHOLED (at a brightness of 1000 cd/m^2^).

The red phosphorescent OLEDs with a mixed host system doped with RD exhibited different device characteristics that depended on the mixing ratios of the two types of hosts. Figure 4(a) shows the current density-voltage-luminance (*J-V-L*) characteristics, and Fig. 4(b) shows the current efficiency as well as the power efficiency as a function of the luminance. The turn-on voltages at a given constant luminance of 1 cd/m^2^ were 4.1, 4.0, 3.8, 3.6, and 3.9 V for **Device A** through **Device E**, respectively. The driving voltage obtained for 1,000 cd/m^2^ of each device were 7.0, 6.6, 5.9, 5.4, and 5.9 V for **Devices A** through **Device E**, respectively. At a given constant luminance of 1,000 cd/m^2^, the current and power efficiencies were 15.3 cd/A and 6.9 lm/W for **Device A**, 17.7 cd/A and 8.4 lm/W for **Device B**, 20.9 cd/A and 10.4 lm/W for **Device C**, 23.4 cd/A and 13.6 lm/W for **Device D**, and 20.9 cd/A and 10.9 lm/W for **Device E**, respectively, as summarized in Table 1. These efficiency data correspond to 12.1%, 14.1%, 16.5%, 19.0%, and 16.8% external quantum efficiencies (EQEs) for **Devices A, B, C, D** and **E**, respectively. The maximum current and power efficiencies were 22.8 cd/A and 11.7 lm/W for Device A, 20.9 cd/A and 13.1 lm/W for **Device B**, 21.9 cd/A and 12.5 lm/W for **Device C**, 25.9 cd/A and 16.3 lm/W for **Device D**, and 22.2 cd/A and 14.6 lm/W for **Device E**, respectively, as summarized in Table 1. Interestingly, the EQE values decreased in the order of **Device D > Device E > Device C > Device B > Device A**. In particular, **Device D** showed the lowest driving voltage of about 5.4 V, a maximum EQE of ~20.7%, and a maximum current efficiency of ~25.9 cd/A which is one of the best efficiency values among those of the solution processed PHOLEDs to date. Moreover, these results are beyond previous data obtained from the devices utilizing same phosphorescent red dopant by evaporation process in terms of the current efficiency, although there are continuous efforts with a great success associated with a new dopant development^27^,^28^.. The reason why **Device E (NH:PH = 10:0)** showed a worse device efficiency as well as a worse driving voltage rather than **Device D (NH:PH = 7:3)** although **Device E** showed better roll-off behavior than **Device D** is due to the biased exciton recombination zone that is close to the HTL/EML interface which causes exciton quenching, especially near the HTL which have relatively low triplet energy (T_1_ : 2.25 eV), while mixing a slight amount of PH shifts the recombination zone toward the EML/B-EBL interface, plausibly due to a suppression of the electron current rather than the hole current after the addition of PH material. In other words, the confinement of the recombination zone inside of the EML (e.g., far away from the HTL/EML and EML/B-EBL interfaces) produces an efficient electroluminescent behavior due to the improved charge balancing effect.

Figure 4(c) shows the EL (electroluminescence) spectra of **Devices A** through **E** at a luminance of 1000 cd/m^2^. Indeed, **Devices A** through **E** exhibited similar EL spectra with a very similar shape with a λ_max_ of 614 nm, presumably due to a comparatively close recombination zone for one another. However, **Devices A** and **B** showed relatively strong side peaks at around 450–520 nm which might have originated from the emission of the common blue layer, as shown in the inset of Fig. 4(c). In particular, this side emission increased in intensity when we increased the portion of the PH because the PH generally causes a bad film morphology with a rough surface and pin-holes, as shown in Fig. 3(a). Thus, we think that the proximity to the pin-holes prompted such a blue emission because some part of the red EML might have been eliminated in these defect sites, resulting in unwanted excition recombination at the blue EML rather than at the red EML. In addition, we also speculate that the recombination zone might have shifted toward blue EML direction in the case of **Devices A** and **B** because the PH more dominantly generates a hole current flow.

In conclusion, we prepared highly efficient solution processed red PHOLEDs with B-EBL and blue common layer structure which could reasonably confines the triplet excitons inside the red EML. The resultant **Device D** with 3:7 ratio of PH:NH doped with 5% of RD gave the highest current and power efficiencies of 23.4 cd/A and 13.6 lm/W at a given luminance of 1000 cd/m^2^. In this condition, we could also obtain the lowest driving voltage of 5.4 V. We found that the relative current density is very useful index to estimate the charge balance for an expectation of highly efficient device composition in case of mixed host systems although many other factors such as surface morphology, solubility, etc. should be also considered for such system.

## Methods

### Materials

We purchased PEDOT:PSS (Clevios^TM^ P VP CH 8000) as a hole injection layer. HL-X026, which is a crosslinking polymer (XP) was purchased from Merck, and used as the hole transporting material without further purification^29^,^30^. NH as the n-type host as well as B-EBL was prepared and purified by standard method^31^. The sublimated grade PH as a p-type host and Ir(mphq)_2_tmd (RD) as a phosphorescent red dopant, BH as a blue host, BD as a blue dopant were also prepared by our previous reports^26^,^27^,^32^. The sublimated grade TPBI as an electron transporting material and LiQ as the organic EIL were purchased from Lumtec Corp. and used without purification. LiF as an inorganic EIL, and Al^33^ as a cathode were purchased from Sigma-Aldrich and also used without purification.

### Device Fabrication

The solution-processed OLED devices were fabricated with 150 nm thick patterned indium-tin oxide (ITO) glasses covered by a bank layer with an open emission area of 4 mm^2^. The ITO glasses were cleaned in acetone and isopropyl alcohol with a sonication process and were rinsed in deionized water. Then, the ITO glass substrates were treated in UV-ozone to eliminate all organic impurities during the previous fabrication processes. PEDOT:PSS (CH8000) was spin-coated as a hole injection layer on ITO glass in an ambient condition and was annealed at 120 °C for 15 min in an inert atmosphere. Subsequently, HTL dissolved in chlorobenzene was spin-coated as a hole transport layer and was crosslinked by using a standard process. In the case of the red EML host, NH and PH materials were dissolved in chlorobenzene to provide a 1 wt% solution, respectively. Then, we mixed both solutions together to obtain five different compostions or various blending ratios. Red EML was spin coated and dried at 100 °C for 10 min. All solution processes were performed in a nitrogen atmosphere at room temperature, except for the PEDOT:PSS. After spin coating with red EML, NH as a B-EBL, blue EML and TPBI were thermally deposited in a vacuum condition under 10^−7^ Torr with 0.5 Å/s. Then, LiF and Al were deposited successively with 0.3 Å/s and 3 Å/s, respectively.

### Measurements

The current density-voltage (*J*–*V*) and luminance-voltage (*L*–*V*) data of the OLEDs was measured by using a Keithley SMU 238 and Minolta CS-100A, respectively. The OLED area was 4 mm^2^ for all samples studied in this work. The electroluminescence (EL) spectra and the CIE coordinates were obtained using a Minolta CS-2000 spectroradiometer.

## Additional Information

**How to cite this article**: Ram Cho, Y. *et al.* Highly efficient organic light emitting diodes formed by solution processed red emitters with evaporated blue common layer structure. *Sci. Rep.* **5**, 15903; doi: 10.1038/srep15903 (2015).

## Figures and Tables

**Table 1:** Device characteristics of the solution processed red PHOLEDs.

Device	Turn-on voltage [V]^a^	Driving voltage [V]^b^	Current Efficiency [cd A^−1^]^b^,^c^	Power Efficiency [lm W^−1^] b,c	EQE [%]^b^,^c^
Device A	4.1 V	7.0 V	15.3 cd/A	6.9 lm/W	12.1%
			(22.8 cd/A)	(11.7 lm/W)	(18.0%)
Device B	4.0 V	6.6 V	17.7 cd/A	8.4 lm/W	14.1%
			(20.9 cd/A)	(13.1 lm/W)	(17.2%)
Device C	3.8 V	5.9 V	20.9 cd/A	10.4 lm/W	16.5%
			(21.9 cd/A)	(12.5 lm/W)	(17.7%)
Device D	3.6 V	5.4 V	23.4 cd/A	13.6 lm/W	19.0%
			(25.9 cd/A)	(16.3 lm/W)	(20.7%)
Device E	3.9 V	5.9 V	20.9 cd/A	10.9 lm/W	16.8%
			(22.2 cd/A)	(14.6 lm/W)	(17.9%)

^a^Measured at 1 cd m^−2^.

^b^Measured at 1000 cd m^−2^.

^c^The parenthesis denotes maximum efficiency.
